# Human J-Domain Protein DnaJB6 Protects Yeast from [*PSI*^+^] Prion Toxicity

**DOI:** 10.3390/biology11121846

**Published:** 2022-12-18

**Authors:** Richard E. Dolder, Jyotsna Kumar, Michael Reidy, Daniel C. Masison

**Affiliations:** 1 Laboratory of Biochemistry and Genetics, National Institute of Diabetes and Digestive and Kidney Diseases, National Institutes of Health, Bethesda, MD 20892, USA; 2 Department of Chemistry, University of Connecticut, Storrs, CT 06269, USA

**Keywords:** DnaJB6, J-domain proteins, amyloid, amyloid toxicity, yeast prions

## Abstract

**Simple Summary:**

DnaJB6 is a human cellular protein quality control factor that prevents diverse peptides from forming disease-associated amyloid, a highly ordered fibrous protein aggregate. DnaJB6 protects human cells, animals and yeast from toxicity caused by amyloids composed of Huntington’s-related polyglutamine, Parkinson’s-related α-synuclein, and ALS- associated TDP-43, and it cures yeast of endogenous prions (infectious amyloids). However, structurally different amyloids of one and the same prion protein, and prions composed of them, can be insensitive to anti-amyloid activity of DnaJB6. This limitation raises concerns about developing DnaJB6 as a therapeutic for amyloid diseases. Prions showing this insensitivity are highly toxic to yeast with reduced function of Sis1, a yeast protein related to DnaJB6. To begin assessing how DnaJB6 might act on amyloid in cells we tested if DnaJB6 could protect yeast from this toxicity. Although it does not eliminate the prion, it protects cells from prion toxicity, but differently than Sis1. Our work provides insight into how human DnaJB6 counteracts cellular toxicity caused by amyloid and establishes an in vivo genetic system useful for studying DnaJB6-amyloid interactions to decipher its mechanisms of action.

**Abstract:**

Human J-domain protein (JDP) DnaJB6 has a broad and potent activity that prevents formation of amyloid by polypeptides such as polyglutamine, A-beta, and alpha-synuclein, related to Huntington’s, Alzheimer’s, and Parkinson’s diseases, respectively. In yeast, amyloid-based [*PSI^+^*] prions, which rely on the related JDP Sis1 for replication, have a latent toxicity that is exposed by reducing Sis1 function. Anti-amyloid activity of DnaJB6 is very effective against weak [*PSI^+^*] prions and the Sup35 amyloid that composes them, but ineffective against strong [*PSI^+^*] prions composed of structurally different amyloid of the same Sup35. This difference reveals limitations of DnaJB6 that have implications regarding its therapeutic use for amyloid disease. Here, we find that when Sis1 function is reduced, DnaJB6 represses toxicity of strong [*PSI^+^*] prions and inhibits their propagation. Both Sis1 and DnaJB6, which are regulators of protein chaperone Hsp70, counteract the toxicity by reducing excessive incorporation of the essential Sup35 into prion aggregates. However, while Sis1 apparently requires interaction with Hsp70 to detoxify [*PSI^+^*], DnaJB6 counteracts prion toxicity by a different, Hsp70-independent mechanism.

## 1. Introduction

### 1.1. Yeast Prions Are Infectious Amyloids

[*PSI^+^*] prions of yeast propagate in the cytoplasm as amyloids composed of the essential translation release factor Sup35/eRF3 [1,2]. Amyloid is a highly ordered, fibrous aggregate of a misfolded protein. [*PSI^+^*] amyloid is infectious, as it is carried with cytoplasm to daughter cells during mitosis, to different strains during mating, and to meiotic progeny during sporulation. [*PSI^+^*] prion phenotypes can vary from weak (*PSI^+^*]^W^) to strong ([*PSI^+^*]^S^), which reflect differences in structures of the Sup35 amyloids of which they are composed. In cells propagating [*PSI^+^*], Sup35 is depleted into insoluble prion aggregates, which reduces its function and causes obvious nonsense suppression phenotypes [3,4]. Strong variants confer stronger nonsense suppression by depleting more Sup35 than weak variants. Despite this depletion, widely studied [*PSI^+^*] prions have little effect on cell growth.

### 1.2. [PSI^+^] Prions Depend on Protein Chaperones for Replication and Have Latent Toxicity

Replication of [*PSI^+^*] prions depends on Hsp104 protein disaggregation machinery that includes Hsp70 and its regulators [5,6]. In attempting to disaggregate prions to resolubilize Sup35, this machinery extracts Sup35 monomers. However, this process causes the amyloid to divide into two smaller fibers that each continue to propagate the prion trait. Sis1 is a class B Hsp40 family J-domain protein (JDP) that regulates Hsp70, is essential for viability, and acts as a crucial regulator of this Hsp104 process [7,8,9,10,11].

Although widely studied [*PSI^+^*] prions have little effect on yeast growth, most [*PSI^+^*] prions are inherently toxic [12,13,14]. Membrane composition can influence amyloid formation by the Sup35 prion determining region [15], but whether this effect is connected to [*PSI^+^*] toxicity is unknown. Well-studied [*PSI^+^*] prions are normally benign, but their inherent toxicity is revealed in cells expressing Sis1JGF, a truncated Sis1 lacking its C-terminal domain (CTD) [16,17,18,19]. The Sis1 CTD, which binds substrates and can interact with the C-terminus of Hsp70, is therefore crucial for Sis1 to protect cells from [*PSI^+^*]^S^ toxicity [19,20]. Reducing Sis1 function or abundance causes prion aggregates to increase in size, which correlates with excessive depletion of Sup35 that causes the toxicity. Thus, in addition to helping promote propagation of [*PSI^+^*], Sis1 moderates toxic depletion of Sup35 in [*PSI^+^*] cells [19].

### 1.3. Human JDP DnaJB6 Protects Yeast from Amyloid Toxicity and Cures Yeast of Prions

Human DnaJB6b is a splice variant of DnaJB6, a JDP related to Sis1 that has a much smaller CTD and an adjacent amyloid-binding serine/threonine-rich (ST) region. DnaJB6b forms oligomers and possesses a potent ST-dependent activity that blocks nucleation and assembly of amyloid by several disease-associated proteins [21,22,23,24,25]. Elevating expression of DnaJB6b protects human cells, yeast, and animals from toxicity of highly expressed Huntington’s related poly-glutamine (polyQ, or polyQ-GFP) [21,24,26], and other human disease proteins, such as alpha-synuclein and TDP-43, which are associated with Parkinson’s disease and amyotrophic lateral sclerosis, respectively [27,28]. 

Toxicity of polyQ-GFP in yeast depends on the presence of [*PSI^+^*] or [*PIN^+^*] prions, which are thought to enhance aggregation of polyQ-GFP that is associated with the toxicity [29,30,31,32,33]. DnaJB6b protects cells from the high toxicity of polyQ-GFP seen in cells propagating both [*PSI^+^*]^S^ and [*PIN^+^*] [24,34]. An interaction of DnaJB6b with Hsp70 is needed for it to sequester disperse polyQ-GFP aggregates into a peri-vacuolar deposition site, but not to detoxify them, showing that the protection does not require sequestration and is probably accomplished directly [34]. The DnaJB6b ST region, which interacts with both the disperse and sequestered polyQ aggregates, and its adjacent CTD are each necessary for the protection. When combined, this ST-CTD (STC) polypeptide is enough to protect cells, but how it detoxifies the polyQ aggregates without noticeably affecting their aggregation state is still unclear.

[URE3] is another yeast prion that propagates as amyloid of Ure2 protein. DnaJB6b prevents purified Ure2 from forming amyloid and it cures yeast of [URE3]. It also prevents Sup35 from forming amyloid structures that produce [*PSI^+^*]^W^ prions when used to infect yeast, and it cures yeast of [*PSI^+^*]^W^ prions. However, it does not prevent Sup35 from forming amyloids structures that propagate as [*PSI^+^*]^S^, nor does it cure cells of [*PSI^+^*]^S^ [24]. These observations reveal a limitation of the anti-amyloid activity of DnaJB6b on structurally different amyloids composed of the same Sup35 protein. Because anti-amyloid activity of DnaJB6 is wide-ranging, these findings surprised us. However, altered versions of DnaJB6b can protect yeast from polyQ-GFP toxicity with minimal effect on polyQ-GFP aggregation state, which suggests that the aggregates do not have to be dismantled or collected to be made non-toxic [34]. Despite its lack of effect on [*PSI^+^*]^S^ prion phenotypes, we hypothesized that DnaJB6 might protect cells from the toxicity of [*PSI^+^*]^S^ observed in cells lacking the Sis1 CTD. Indeed, although we do not observe anti-prion activity of DnaJB6b when intact Sis1 is present, we find it can reduce toxicity of [*PSI^+^*]^S^ in cells expressing Sis1JGF in place of Sis1. DnaJB6b also prevented excessive aggregation of Sup35 upon depletion of Sis1, and this protection occurs with only mild effects on prion strength and stability. Our data show that DnaJB6b can protect yeast from toxicity of [*PSI^+^*] prions by moderating depletion of Sup35, like Sis1, but by a seemingly different mechanism.

## 2. Materials and Methods

### 2.1. Yeast Strains, Plasmids, and Growth Conditions

Yeast strains are isogenic to wild type strain 779-6A (*MATa, kar1*, *SUQ5* (*SUP16*), *ade2-1*, *his3*, *leu2*, *trp1*, *ura3* [3]). All are [*pin^−^*]. Strain 628-8Cc, used for cytoduction, is described [18]. 970L is [*psi^−^*], *sis1∆/*pYW17 (*URA3*, *SIS1*), pRS315sis1JGF (*LEU2*, *sis1JGF*). 970L^S^, used for plasmid shuffles, is the same but propagates the strong [*PSI^+^*] variant [*PSI^+^*]^S^. Strain 2140 is *sis1∆*, *NGMC* (Sup35-GFP [35]) and has a *URA3* plasmid encoding *SIS1* regulated by a tetracycline repressible promoter. This plasmid, named pRED156, is pRS316 (*CEN6*, *URA3* [36]) with its *Eco* RI—*Not* I fragment replaced by the *Eco* RI—*Not* I fragment from pTETrSIS1 [10]. Plasmid pJE237 and pJE65 were made by subcloning the *Bam* HI—*Sal* I fragment from pYW62 [37] into the same sites in pRS315 and pRS314, respectively. These and remaining plasmids are listed in Table 1.

Standard growth media and conditions were used [39]. Excess adenine (400 mg/L) was added to all liquid media. Limiting amounts of adenine (10–20 mg/L) were used for solid media only when monitoring prions. Cells were grown at 30° unless indicated otherwise. For Sis1 depletion experiments doxycycline was added to cultures at a final concentration of 6 μg/mL.

### 2.2. Monitoring Prions

Sup35 promotes efficient translation termination by facilitating release of nascent polypeptides when ribosomes encounter stop codons. The presence of [*PSI^+^*] reduces translation termination by depleting Sup35 into insoluble amyloid aggregates, causing a nonsense suppressor phenotype. In the absence of [*PSI^+^*], termination of translation at the premature stop codon of *ade2-1* mediated by Sup35 is efficient, which makes our strains Ade- and when grown on limiting adenine they are red. The combination of [*PSI^+^*]^S^ and *SUQ5* in our strains suppresses *ade2-1* enough to make cells Ade+ and white. [*PSI^+^*]^W^ prions deplete less soluble Sup35, which produces a “weaker” intermediate pink color.

Cytoductions were done as described [40] using recipient strain 628-8Cc to confirm presence of prions in cells and to determine if effects of JDPs on prions could be due to alteration of physical properties of the underlying amyloid. Defective nuclear fusion in *kar1* cells after mating results in progeny that share cytoplasm but retain their original genotypes. By this abortive mating “Donor” cells with prions infect 628-8Cc cells. Prion phenotype of infected recipients reports on the prions present in the donor.

### 2.3. Plasmid Shuffle

Plasmid shuffle was done as described [19]. Briefly, strain 970L^S^ expresses Sis1JGF from a *LEU2* plasmid to provide Sis1 function needed to support viability. Sis1JGF cannot counteract toxicity of [*PSI^+^*]^S^, so 970L^S^ also carries a *URA3* plasmid encoding Sis1 that protects cells from this toxicity. The presence of this plasmid (pYW17) makes cells sensitive to 5-fluoro-orotic acid (FOA), which kills Ura+ cells. 970L^S^ was transformed by *TRP1* plasmids encoding proteins to be tested for ability to counteract [*PSI^+^*]^S^ toxicity selecting on medium containing uracil, which allows loss of the *URA3* plasmid. Individual transformants were grown as patches of cells on similar plates, and then replica-plated onto similar medium containing FOA. Images of plates were taken after 2–4 days incubation at 30°. *CEN* plasmids mis-segregate at low frequency (~2%) during mitosis and cells that fail to inherit pYW17 will not grow on FOA unless the test protein can protect from prion toxicity in place of Sis1. Density of growth on FOA provides an initial estimate of this protection. 

### 2.4. Growth Rates

Cells in SC medium selecting for plasmids were grown in 24-well microtiter plates shaking at 300 rpm in a microplate reader. OD_600_ was measured every 20 min for 48 h. Cells were grown in 1 mL of total volume starting at OD_600_ ~ 0.02. 

### 2.5. Western Blots for Sis1 Abundance

Abundance of Sis1 was determined by Western blot as described [19] using log phase cells grown 28 h (~7 generations) in YPAD, or selecting medium when necessary, with or without doxycycline (6 μg/mL). Briefly, 10 μL of lysates (at OD_600_ = 0.6) were separated by 4–20% SDS-Page Criterion^TM^TGX^TM^ gels. Proteins were transferred to PVDF membranes and probed with Sis1 antibodies [19] diluted 1:10,000 and HRP-conjugate anti-rabbit secondary antibodies diluted 1:6000. Amounts of Sis1 were quantitated using Image-J. 

### 2.6. Fluorescence Microscopy

Strain 2140 log phase cells were grown in SC medium selecting for plasmids and fixed via paraformaldehyde (http://mcb.berkeley.edu/labs/koshland/Protocols/MICROSCOPY/gfpfix.html, accessed on 3 October 2022) before, and 28 h after 6 μg/mL of doxycycline was added. Control cells were grown similarly without doxycycline. Microscopic images were captured on a Nikon E-800 microscope using Nikon NIS Elements software (04.30.02 64 bit, Nikon Instruments Inc., New York, NY, USA) with a Q-Imaging Retiga EXi digital camera, Plan APO VC 60X oil immersion DIC optics, GFP and RFP filters. Images were processed for presentation using Adobe Photoshop 2022 software.

## 3. Results

### 3.1. DnaJB6b Protects Yeast from Toxicity of [PSI^+^]^S^ Prions

**Figure 1:**
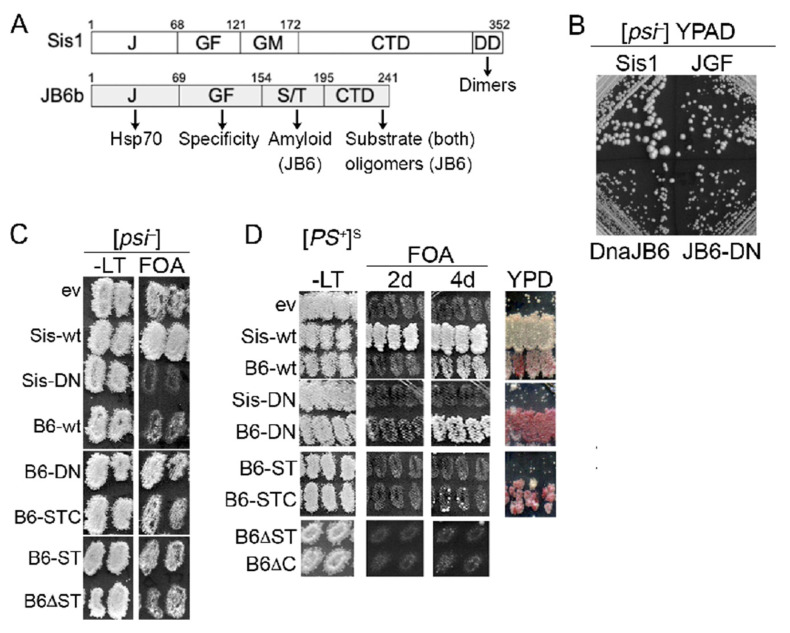
DnaJB6 protects yeast from toxicity of [*PSI^+^*]^S^. (**A**) Structures of Sis1 and DnaJB6b (JB6b) drawn near to scale. Numbers indicate amino acid residues that delineate structural domains. J-domain mediates interaction with Hsp70, GF (DnaJB6) and GF/GM (Sis1) confer functional specificity, ST of DnaJB6 interacts with amyloid, and CTD binds misfolded protein substrates. Terminal CTD region of Sis1 encodes a dimerization region (DD); CTD of DnaJB6 is important for forming higher-order oligomers [25]. (**B**) FOA-resistant [*psi^−^*] cells expressing Sis1JGF only or Sis1JGF plus indicated proteins were grown on rich medium for 3 days at 30° and 1 day at 22°. (**C**) Plasmid shuffle (see text) using strain 970L^S^. All are *sis1∆* and express Sis1 from a *URA3* plasmid, Sis1JGF from a *LEU2* plasmid and test protein indicated on left from a *TRP1* plasmid. Cells grown on SC -Leu-Trp (-LT) were replica-plated onto similar medium containing FOA and grown 2 days. (**D**) Same as (**C**) but [*PSI^+^*]^S^ cells. FOA plate was incubated 2–4 days as indicated at top. FOA plate was replica-plated onto 1/2YPD (YPD), which has limiting amount of adenine to monitor prion color phenotype. For (**C**,**D**); ev, empty *TRP1* vector (Sis1JGF alone); B6, DnaJB6b.

Sis1JGF, a truncated Sis1 that contains only the J and GF domains (amino acids 1–121) and lacks the substrate-binding CTD (see Figure 1A), provides the functions of Sis1 needed for cell viability. When expressed as the only source of Sis1, however, Sis1JGF cannot prevent toxicity of [*PSI^+^*]^S^ (a strong variant of [*PSI^+^*]) caused by depletion of functional Sup35 protein into prion aggregates [16,19]. Therefore, in *sis1∆* cells (lacking Sis1) that express Sis1JGF and propagate [*PSI^+^*]^S^, we co-express full-length Sis1 from a plasmid to keep enough Sup35 functional for cells to grow. JDPs have a conserved HPD (His-Pro-Asp) motif that mediates interaction with Hsp70, and homologous mutations of the aspartate of this motif (D36N in Sis1 and D33N in DnaJB6b) disrupt interaction with Hsp70 [41]. Sis1-D36N cannot support growth in place of Sis1, indicating that interaction of Sis1 with Hsp70 is essential for viability. DnaJB6b cannot support growth of *sis1∆* cells [24], so it is not a functional homolog of Sis1.

To test if intact or mutant versions of DnaJB6b could counteract toxicity of [*PSI^+^*]^S^ when expressed in place of Sis1 we used a “plasmid shuffle” assay to exchange *TRP1* plasmids encoding test proteins for a *URA3* plasmid encoding Sis1 (pYW17) [19]. Our *sis1∆* strain 970L^S^ expresses Sis1JGF from a *LEU2* plasmid and Sis1 from pYW17. Selection for the plasmid encoding Sis1JGF is maintained to support growth when pYW17 is lost, but pYW17 cannot be lost unless a test protein exchanged for Sis1 can protect cells from [*PSI^+^*]^S^ toxicity. We monitor whether pYW17 can be lost using plates containing FOA, which kills Ura^+^ cells. Cells from FOA that lost pYW17 will depend on Sis1JGF to provide essential Sis1 functions. They also depend on a version of DnaJB6b that counteracts [*PSI^+^*]^S^ toxicity, so selection is not needed for either plasmid after recovery from FOA.

Expressing DnaJB6b slows growth of wild type yeast [34], so we first performed the plasmid shuffle using [*psi^−^*] cells (strain 970L) to determine if expression of any other test protein affected growth in the absence of prions. Several individual transformants of each test protein were isolated from primary selection plates (SC -Leu-Trp) and inoculated as patches onto a master plate of similar medium. These were then replica-plated onto similar medium containing FOA to select for cells having lost pYW17.

Although [*psi^−^*] FOA-resistant cells that express only Sis1JGF (ev) grow more slowly than those expressing wild type Sis1 (Figure 1B), they are evident on FOA plates after incubating two days (Figure 1C). FOA-resistant [*psi^−^*] cells expressing Sis1JGF with DnaJB6b or DnaJB6b-D33N in place of Sis1 also grew on FOA, but more slowly than those expressing Sis1JGF alone (Figure 1B,C, Table 2). While those co-expressing DnaJB6b-D33N grew on FOA to a similar density as those expressing Sis1JGF alone, those expressing DnaJB6b took four days to show appreciable growth on FOA (Figure 1C).

We quantified growth rates of [*psi^−^*] cells recovered from FOA and found that, compared with cells expressing Sis1JGF alone, the doubling time of cells expressing DnaJB6b was 13% (45 min) longer, while that of cells expressing DnaJB6b-D33N was 5% (15 min) longer (Table 2). These results suggest that an Hsp70-independent activity of DnaJB6b reduces growth and that binding of DnaJB6b to Hsp70 further inhibits growth, perhaps by competing with endogenous co-chaperones for interaction with Hsp70.

Surprisingly, although FOA-resistant [*psi^−^*] cells with Sis1JGF and the empty *TRP1* vector were readily recovered in 2 days, we failed to recover viable [*psi^−^*] cells expressing both Sis1JGF and Sis1-D36N after a week. These results suggest that Sis1-D36N interferes with the ability of Sis1JGF to support growth even in cells without prions.

For the plasmid shuffle using [*PSI^+^*]^S^ cells (strain 970L^S^), the rate of growth on FOA and after recovery from FOA generally reflects the extent a test protein can protect from prion toxicity in the absence of Sis1. In line with our earlier findings, [*PSI^+^*]^S^ transformants with the empty *TRP1* vector (i.e., expressing only Sis1JGF) were not recovered on FOA, while control cells with *SIS1* on the *TRP1* plasmid showed strong confluent growth on FOA after incubating for a day. As with [*psi^−^*] cells, [*PSI^+^*]^S^ cells expressing Sis1-D36N did not grow on FOA, indicating they were dependent on wild type Sis1 for viability. (Figure 1D).

[*PSI^+^*]^S^ transformants expressing DnaJB6b grew on FOA, although we did not observe appreciable growth until plates were incubated for four days. Thus, although DnaJB6b is itself somewhat toxic, it alleviated toxicity of [*PSI^+^*]^S^. DnaJB6b-D33N also counteracted [*PSI^+^*]^S^ toxicity. Consistent with the [*psi^−^*] cells, [*PSI^+^*]^S^ cells expressing DnaJB6b-D33N grew faster than those with wild type DnaJB6b, showing noticeable growth after 2 days and denser growth after 4 days. These results show that DnaJB6b can counteract toxicity of [*PSI^+^*]^S^ independently of Hsp70.

To gauge the extent that DnaJB6b was inhibiting propagation of [*PSI^+^*]^S^ in cells lacking the Sis1 CTD, we monitored prion status of transformants on plates with limiting adenine (1/2YPD), where our [*psi^−^*] cells are red. The amount of red pigmentation in [*PSI^+^*]^S^ cells reflects the amount of Sup35 that remains soluble [3,4], and thus the extent that test proteins reduce incorporation of Sup35 into prion aggregates. Mitotic stability is another prion characteristic, typically linked to the number of prion “seeds” per cell that can be distributed between dividing cells. Cells with fewer seeds have a higher chance of giving rise to daughters that fail to inherit prions during cell division and become [*psi^−^*]. 

As an initial assessment for prion phenotypes, we replica-plated cells from the FOA plates onto 1/2YPD. Replica-plated patches of cells co-expressing DnaJB6b or DnaJB6b-D33N together with Sis1JGF had obvious red coloration (Figure 1D). Thus, both impaired propagation of [*PSI^+^*]^S^. DnaJB6b-D33N caused a redder prion phenotype, indicating its anti-prion effect was stronger.

**Figure 2:**
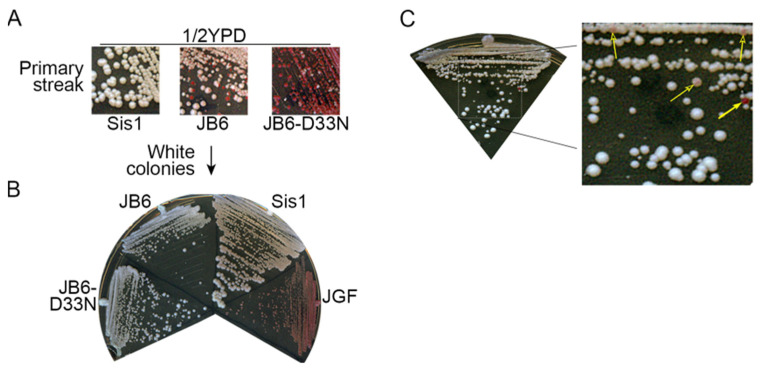
DnaJB6b impairs [*PSI^+^*]^S^ propagation weakly. (**A**) Cells recovered from FOA plates of the plasmid shuffle in Figure 1D were streaked onto 1/2YPD medium to monitor prion status—red cells are [*psi^−^*]. Cells expressing DnaJB6b (JB6) or DnaJB6b-D33N (JB6-D33N) are mixtures of red and white colonies. (**B**) Cells from white colonies in panel (**A**) produce only white colonies when re-streaked. Cells lacking prions and expressing Sis1JGF in place of Sis1 are shown as [*psi^−^*] control. (**C**) Continued expression of DnaJB6b in cells with Sis1JGF in place of Sis1 lose prions at low frequency, producing rare red colonies (filled arrow, [*psi^−^*]) and pink colonies (open arrows, mixtures of red [*psi^−^*] and white [*PSI^+^*]^S^ give pink color).

The pink color of patches or colonies could reflect a weakening of the prion in all cells, a change of the prion variant, or a mixture of red cells that have lost the prion and white cells that have a normal prion phenotype. Finding red colonies among white ones would indicate that DnaJB6b-D33N, and perhaps other test proteins, can cure cells of the prion, which would allow cells without [*PSI^+^*] to grow on FOA without Sis1 or DnaJB6b. To test if any cells were [*psi^−^*] we first took them from the FOA plates and streaked them to single colonies on similar plates. All transformants with pink to red color gave rise to mixtures of red and white colonies (Figure 2A). 

Subsequent streaking showed cells from red colonies produced only red colonies and those from white colonies gave rise to only white colonies (Figure 2B). These results indicate that red cells had lost [*PSI^+^*]^S^ and white cells propagated [*PSI^+^*]^S^ stably. Thus, DnaJB6b and DnaJB6b-D33N counteracted [*PSI^+^*]^S^ toxicity while reducing [*PSI^+^*]^S^ strength or stability only modestly. Apparently, their inhibitory effect on [*PSI^+^*]^S^ was enhanced during the plasmid shuffle process. We note that upon continued re-streaking of [*PSI^+^*]^S^ cells expressing DnaJB6b, rare red colonies and pink colonies arose among the mostly white ones (Figure 2C). This observation is consistent with a modest ability of DnaJB6 to weaken [*PSI^+^*]^S^ prions. In contrast, extra Sis1 did not weaken [*PSI^+^*]^S^ prion phenotype or cause any detectable loss of [*PSI^+^*]^S^ during continued subculturing (Figure S1A). Taken together, our data show that modest impairment of prion propagation is enough to protect cells from [*PSI^+^*]^S^ toxicity and suggest that the ways DnaJB6b and Sis1 counteract toxicity of [*PSI^+^*]^S^ are different.

The STC polypeptide was unusual in that rather than providing general protection of all cells, only 4–10 colonies arose on the FOA plates of the replica-plated patches of STC transformants (Figure 1D). While one or two FOA-resistant colonies arose among dozens of transformants of the empty vector, this pattern was evident in six of nine STC transformants, which suggests the higher frequency of FOA-resistant cells required expression of STC. These FOA-resistant colonies also were mixtures of red and white cells, showing the STC region is enough to disrupt [*PSI^+^*]^S^ propagation without affecting nonsense suppression. The very low frequency at which cells were recovered from FOA, however, raises genuine concerns that an additional effect was allowing STC to help these rare cells survive. We therefore did not continue using STC cells recovered from FOA for this study.

To assess if the test proteins that counteracted prion toxicity might have changed some physical property of the Sup35 amyloid to create a new prion variant, we used cytoduction, an abortive mating, to transmit prions to a wild type [*psi^−^*] strain (see Methods). All recipients infected by [*PSI^+^*] donors, including the wild type Sis1 control, had indistinguishable white and mitotically stable [*PSI^+^*]^S^ phenotypes (Figure S1B). Thus, the DnaJB6b test proteins were weakening toxicity of [*PSI^+^*]^S^ without changing the prion to a weaker or less stable variant.

### 3.2. DnaJB6b Prevents Hyper-Aggregation of Sup35 When Sis1 Is Depleted

To determine whether observed effects on counteracting [*PSI^+^*]^S^ toxicity were associated with reduced depletion of Sup35 into prion aggregates, we monitored Sup35 aggregation status using a functional GFP-tagged version of Sup35 (called NGMC), which has GFP inserted between the N and M domains of Sup35 [35]. In [*psi^−^*] cells, NGMC shows diffuse fluorescence, but in [*PSI^+^*] cells NGMC forms many small disperse foci [42,43]. We refer to NGMC prions as [*GPSI^+^*] to distinguish them from prions composed of untagged Sup35 because they have a very faint pink color phenotype, which indicates they are slightly weaker prions [35]. Accordingly, we find [*GPSI^+^*]^S^ are toxic, but not lethal, in cells expressing Sis1JGF in place of Sis1 (Figure S2). Earlier we showed that depleting Sis1 by excising the *SIS1* gene from a plasmid in *sis1∆* [*GPSI^+^*]^S^ cells causes [*GPSI^+^*] aggregates to become strikingly larger and brighter [19]. This hyper-aggregation coincides with reduced solubility of NGMC (i.e., Sup35). Sis1JGF cannot moderate this excess incorporation of NGMC into the prions, suggesting the CTD of Sis1 is needed to help [*PSI^+^*]^S^ cells survive by keeping enough Sup35 soluble [19]. 

Our finding that DnaJB6b counteracts [*PSI^+^*]^S^ toxicity suggests it also might moderate incorporation of Sup35 into prion aggregates. Here, we used a doxycycline repressible *TETr* promoter system to deplete Sis1 [10]. Our *sis1∆* strain 2140 expresses NGMC in place of Sup35, it propagates [*GPSI^+^*]^S^, and it expresses Sis1 from the *TETr* promoter on a single-copy *URA3* plasmid. Addition of doxycycline to cultures represses transcription from this promoter, and Sis1 is subsequently depleted as cells continue to grow. This strain does not have Sis1GF on the *LEU2* plasmid. Test proteins were again expressed from *TRP1* plasmids.

**Figure 3:**
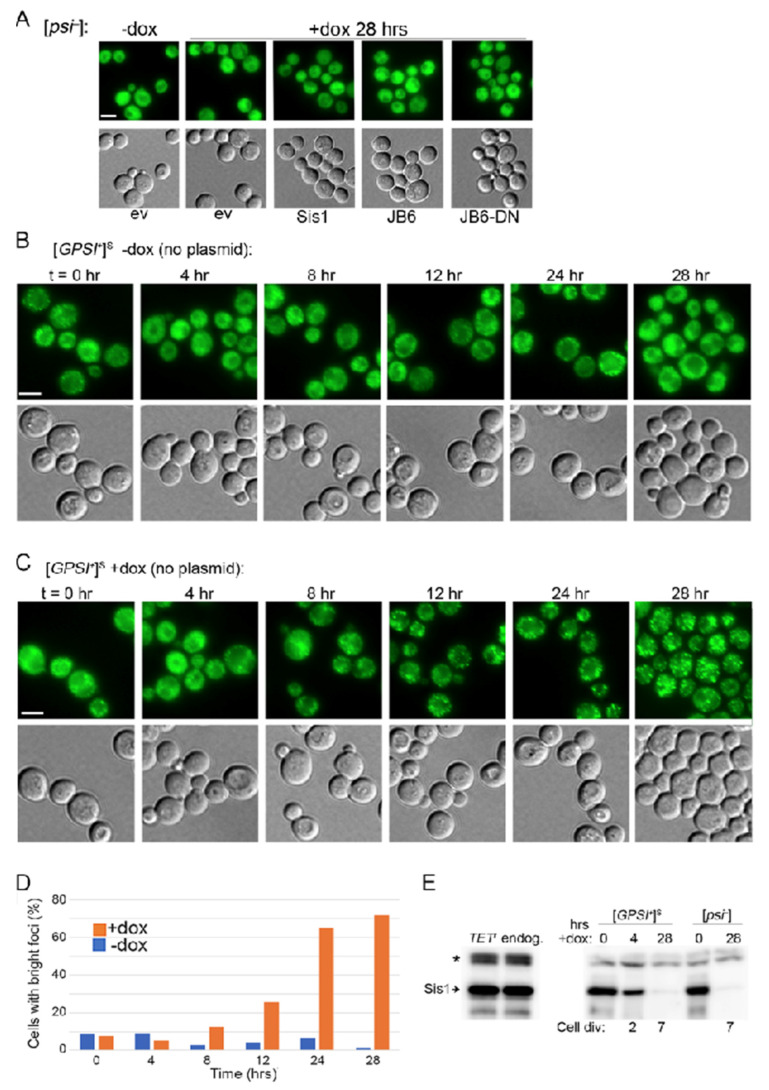
Depleting Sis1 causes hyper-aggregation of NGMC in [*PSI^+^*] cells of strain 2140. (**A**) NGMC fluorescence is diffuse in the cytoplasm of [*psi^−^*] cells with or without doxycycline, or with plasmids that express the test proteins or carry empty vector (ev) indicated below. (**B**) NGMC fluorescence over time for [G*PSI^+^*]^S^ cultures without doxycycline. (**C**) Same as in panel (**B**) but doxycycline was added at time t = 0. (**D**) Quantitation of percent of cells in population in panel (**B**) that have large bright aggregates; (*n* = 100–200). (**E**) Western blot probing for Sis1 expressed from *TETr* or endogenous promoter (*left*, asterisk indicates cross-reacting bands useful as load control); and in [*PSI^+^*]^S^ and [*psi^−^*] cells exposed to doxycycline for indicated times (*right*). Numbers below indicate number of cell divisions. Scale bars = 5 μm; all images in each panel are at same scale.

We first assessed if depleting Sis1 by using the *TETr* system affected fluorescence of NGMC in a [*psi^−^*] version of strain 2140. In untreated cultures, NGMC fluorescence was consistently diffuse in the cytoplasm of most cells, indicating it was soluble (Figure 3A). This pattern was unchanged by treatment with doxycycline for 28 h (~7 generations), or when the empty vector or any of the test proteins was also present. Thus, the status of soluble NGMC protein was not affected by the depletion of Sis1 or the presence of any test protein.

For [*GPSI^+^*]^S^ cells we first monitored NGMC fluorescence in cells without test proteins periodically during the 28 h treatment. Without doxycycline, NGMC formed many small disperse foci that represent prion aggregates in a background of diffuse fluorescence (Figure 3B). This pattern did not change throughout the time course. After adding doxycycline to [*GPSI^+^*]^S^ cultures the size and brightness of NGMC foci began increasing at about 8 h and continued until 28 h when the increase in brightness and number of foci tapered off (Figure 3C,D). This increase in aggregate size coincided with a noticeable reduction in the diffuse background fluorescence, which suggests the foci are incorporating cytosolic Sup35.

Earlier work by others shows the amount of Sis1 in doxycycline-treated cultures drops below detection by 18 generations, yet cultured cells continue to grow for over a hundred generations and to propagate [*PSI^+^*] stably for 30 generations [11]. As Sis1 is essential for viability [7], trace amounts of Sis1 are enough to support cell growth and [*PSI^+^*] propagation, and presumably to protect from [*PSI^+^*] toxicity. To confirm effectiveness of doxycycline repression of Sis1 expression in our hands, we monitored abundance of Sis1 by Western blot (Figure 3E). Exposure to doxycycline for 28 h reduced the amount of Sis1 to less than 3% of that of untreated cells, which is comparable to earlier data [10,44].

**Figure 4:**
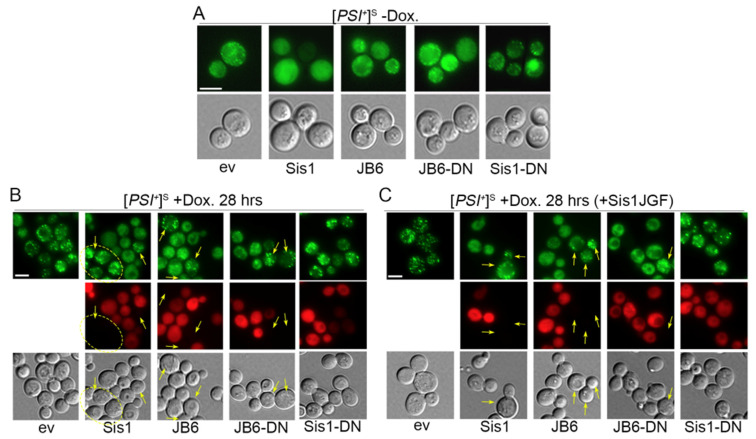
DnaJB6b prevents hyper-aggregation of NGMC in strain 2140 when Sis1 is depleted. (**A**) [G*PSI^+^*]^S^ cells express the indicated test proteins or carry an empty vector (ev). Cells with normal amount of Sis1 (ev) have noticeable NGMC foci. Extra Sis1 causes disappearance of foci, expression of other test proteins does not. (**B**) Cells were treated with doxycycline for 28 h. Those expressing Sis1JGF alone (ev) have large bright foci. Those co-expressing Sis1-mCherry, DnaJB6b-mCh (JB6) or DnaJB6b-D33N-mCh (JB6-DN) have mixtures of cells with and without large foci. All cells that lack large foci express test protein (red), while all those that show large foci (arrows) do not. Expression of Sis1-D36N does not prevent hyper-aggregation of NGMC. (**C**) Experiment repeated using cells expressing Sis1JGF from a *LEU2* plasmid. Scale bars = 5 μm; all images in each panel are at same scale.

Anti-amyloid activity of DnaJB6b is associated with its ST region binding to amyloid [23,24,26,45]. Our finding that DnaJB6b∆ST did not protect cells from prion toxicity is consistent with protective activity of DnaJB6b being linked to its binding to Sup35 prion amyloid. To test this hypothesis, we fused mCherry (mCh) to the C-terminus of Sis1, DnaJB6b and DnaJB6b-D33N so we could assess their co-localization with NGMC. Recovery of 970L^S^ transformants expressing these tagged proteins on FOA was the same as their untagged counterparts (Figure S3), showing the mCh tag had little effect on ability of the proteins to protect cells from [*PSI^+^*]^S^. Expressing DnaJB6b-mCh or DnaJB6b-D33N-mCh in [*GPSI^+^*]^S^ cells without doxycycline had little effect on the pattern of NGMC foci (Figure 4A). In contrast, an extra *SIS1* gene on the *TRP1* plasmid (encoding Sis1-mCh) caused the foci to disappear and the fluorescence to become completely diffuse. We saw no visible difference in color phenotype of cells with one or two wild type *SIS1* genes and, despite their [*psi^−^*] like fluorescence, they were not cured of [*GPSI^+^*]^S^. These data suggest that doubling the expression of Sis1 in otherwise wild type cells was reducing incorporation of Sup35 into prion aggregates without noticeably reducing prion-dependent nonsense suppression or prion replication. For all test proteins, the red fluorescence was diffuse in the cytoplasm with no visible foci. These data suggest they did not co-localize with the small NGMC foci, but at this resolution we cannot make this conclusion with certainty.

To test if DnaJB6b-mCh could reduce hyper-aggregation of NGMC or co-localize with the larger NGMC foci when Sis1 is depleted, we treated 2140 [*GPSI^+^*]^S^ cells expressing DnaJB6b-mCh or DnaJB6b-D33N-mCh with doxycycline as above and monitored fluorescence (Figure 4B). As controls we used Sis1-mCh and an empty vector. Roughly 9% of cells with the empty vector had the large NGMC foci even before adding doxycycline to repress Sis1 expression. This proportion likely represents a combination of cells that have lost the *TETr* plasmid and those that otherwise fail to express Sis1. After 28 h of treatment with doxycycline that proportion increased to 97%.

Cells co-expressing Sis1-mCh, DnaJB6b-mCH, or DnaJB6b-D33N-mCh with the *TETr*-regulated Sis1 did not form the large NGMC foci (Figure 4B). In these cells the foci looked much like the smaller foci in cells with the wild type amount of Sis1 (Figure 4A, ev). For each of these strains the red fluorescence was diffuse, again preventing confident assessment of co-localization. There was cell-to-cell variation in expression of each test protein as indicated by differences in intensity of red fluorescence, and each had a fraction of cells with no detectable red fluorescence, indicating the test protein was not expressed. We suspect these differences in fluorescence intensity were due to gain or loss of plasmids during mitosis.

Conspicuously, we did not observe the large NGMC foci in any of the cells showing any detectable red fluorescence but did see them in all non-red cells. This complete correlation implies that Sis1, DnaJB6b and DnaJB6b-D33N each actively prevented the formation of large NGMC foci caused by depleting Sis1 with doxycycline treatment. This effect correlated with reduced toxicity, suggesting toxicity is counteracted by limiting incorporation of NGMC into prion aggregates, which agrees with earlier data linking inhibition of hyper-aggregation with increased Sup35 solubility [19]. 

Untreated [*GPSI^+^*]^S^ cells with a normal amount of Sis1 that also expressed Sis1-D36N-mCh had a typical punctate pattern of NGMC fluorescence (Figure 4B), unlike the smooth and diffuse NGMC fluorescence seen in cells with the extra copy of Sis1 (Figure 4A). Because Sis1-D33N can form dimers with wild type Sis1, these results suggest that the ability of extra of Sis1 to reduce the size of [*PSI^+^*]^S^ prion aggregates required normal J-domain interaction with Hsp70. After depleting Sis1 in these cells with doxycycline, large prion aggregates formed in 90% of red-fluorescent cells expressing Sis1-D36N-mCh (Figure 4B). Again, these results suggest that, unlike DnaJB6b, interaction of Sis1 with Hsp70 is needed for it to moderate incorporation of Sup35 into prion aggregates.

Although Sis1-D36N prevented growth of cells expressing Sis1JGF in place of Sis1, it did not slow growth of [*PSI^+^*]^S^ or [*psi^−^*] cells expressing typical amounts of wild type Sis1. Moreover, we found it took 48 h (~12 generations) of growth in doxycycline before Sis1 was sufficiently depleted for toxic effects of Sis1-D36N to be observed. For all images in Figure 4B, cells were grown for 28 h (~7 generations) in doxycycline. Thus, the formation of large NGMC foci in cells expressing Sis1-D36N was linked to its inability to prevent the hyper-aggregation and not to toxicity of Sis1-D36N. We repeated the experiment in Figure 4B with all cells co-expressing Sis1JGF and obtained essentially identical results (Figure 4C), showing Sis1JGF did not enhance or hinder formation of larger aggregates and that preventing the hyper-aggregation depended on the CTD of Sis1.

### 3.3. NGMC Can Be Recovered from Large Prion Aggregates

**Figure 5:**
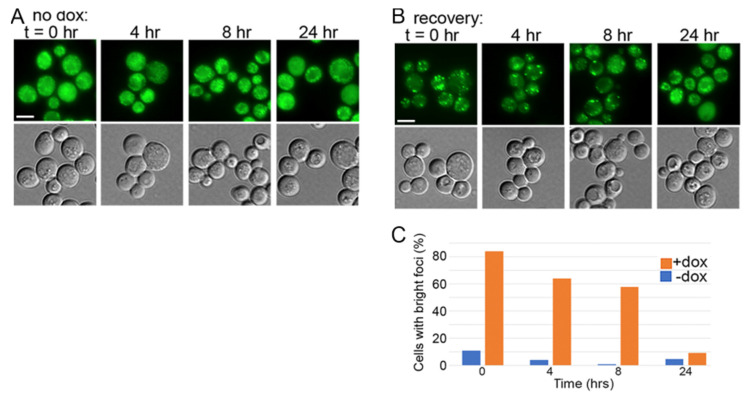
NGMC is recovered from large aggregates by restoring Sis1. (**A**) Cells of strain 2140 grown without doxycycline for 28 h and then monitored for fluorescence at the times indicated above. (**B**) Parallel cultures grown with doxycycline for 28 h and then monitored similarly after switching to medium without doxycycline. (**C**) Quantitation of percent of cells in population in panel B, that have large bright aggregates (*n* = 100–200). Scale bars = 5 mm; all images in each panel are at same scale.

To assess if the larger aggregates were irrecoverable dead-end products or could be resolubilized by restoring Sis1 expression, we transferred doxycycline-treated cells to fresh medium without doxycycline and monitored the status of NGMC fluorescence. After 8 h we began to see a reduction in the number of cells with large foci. By 24 h (~5 generations) only 9% of cells had very bright foci and in the remaining cells the foci and background fluorescence resembled that of untreated cells (Figure 5A,B). After recovering for 24 h the cells that still had bright foci, possibly having lost the plasmid, had a typical number of brighter foci and lower background fluorescence, suggesting that the foci were not simply being diluted among the growing number of cells. These results suggest that solubility of Sup35 was increasing as NGMC was recovered from the foci and that restoring Sis1 was primarily responsible for this recovery.

## 4. Discussion

DnaJB6b very effectively cures yeast of [*PSI^+^*]^W^ prions and in vitro it blocks formation of Sup35 amyloid that composes [*PSI^+^*]^W^ prions, but it does not affect [*PSI^+^*]^S^ prions or formation its underlying Sup35 amyloid [24]. These findings show that the potent and broad anti-amyloid activity DnaJB6b has limitations. Nevertheless, when Sis1 function or abundance is compromised, our finding that DnaJB6b reduces prion toxicity shows it can weaken [*PSI^+^*]^S^ to some degree. The [*PSI^+^*]^S^ Sis1JGF system therefore provides a sensitive approach to detect protective anti-amyloid effects of DnaJB6b in vivo on a structural variant of amyloid that resists elimination.

The curing of [*PSI^+^*]^S^ by continued expression of DnaJB6b in cells with Sis1JGF in place of Sis1, although very inefficient, is further evidence of its anti-[*PSI^+^*]^S^ activity in cells. The ability of DnaJB6b to counteract prion toxicity depended on its ST region, which contains the amyloid-binding properties of DnaJB6, suggesting it acts directly. This interpretation is further supported by our finding that the D33N mutant, which does not interact with Hsp70, protects from [*PSI^+^*]^S^ prion toxicity as effectively as intact DnaJB6b.

**Figure 6:**
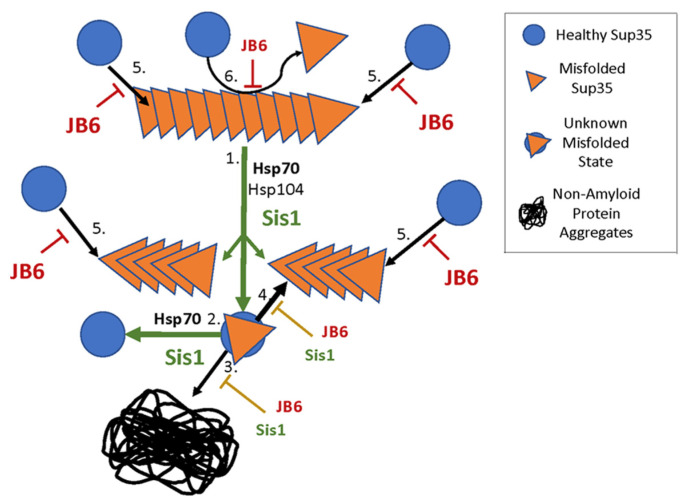
Possible ways that Sis1 and DnaJB6 (JB6) help keep Sup35 soluble in [*PSI^+^*]^S^ cells. The blue circles represent properly folded Sup35. The orange triangles represent misfolded [*PSI^+^*]^S^ Sup35. The stacked orange triangles represent Sup35 prion amyloid sheets that grows by adding Sup35 monomer to its ends and converting them to amyloid as they join the fiber (arrows 5). Green arrows show our proposed primary function of Sis1, which is to cooperate with Hsp70 and Hsp104 extract Sup35 monomers out of the amyloid sheet thereby dividing it into two smaller amyloids (arrow 1) that can continue to grow. The extracted protein represented by the overlapped circle and triangle is in an unknown misfolded state that has three different fates as shown by arrows 2–4. Sis1 with Hsp70 could refold Sup35 to its native conformation (arrow 2). The misfolded protein could aggregate in a non-amyloid manner (arrow 3), or the protein could re-join the amyloid, perhaps more readily than native Sup35 (thicker arrow 4). By promoting refolding (arrow 2), Sis1 would reduce arrows 3 and 4. Sup35 is capable of binding to the ends of prion amyloid at any point (arrows 5). Sup35 likely interacts with the surfaces of the amyloid, where secondary nucleation might also occur (arrow 6). We propose DnaJB6 (JB6) mainly acts by inhibiting arrows 3–6 via binding directly to the amyloid or aggregate, thus sterically hindering its interaction with native Sup35. Sis1 needs to interact with Hsp70 to help keep Sup35 soluble, so we suspect it mainly acts through arrows 1 and 2.

Because moderating depletion of Sup35 in [*PSI^+^*] cells is the main factor in preventing [*PSI^+^*] toxicity, our data suggest that DnaJB6b, like Sis1, prevented toxic depletion of Sup35. However, expressing Sis1-D36N in wild type cells did not reduce size of prion aggregates like wild type Sis1 and it failed to prevent the hyper-aggregation seen when Sis1 is depleted. Unlike DnaJB6b, these results indicate the J-domain interaction of Sis1 with Hsp70 was important for these activities. Moreover, Sis1 does not possess an ST region, which DnaJB6 needed to counteract prion toxicity. Together our data point to a difference in the ways these JDPs counteract prion toxicity. Figure 6 illustrates possible ways Sis1 and DnaJB6b moderate prion-mediated depletion of Sup35.

Sis1 could cooperate with Hsp70 to keep Sup35 soluble by helping the monomers that are extracted from prion fibers by the Hsp104 machinery refold into a native conformation. This action could make them less prone to be captured by the amyloid template, to be depleted into non-amyloid aggregates or to be degraded. As DnaJB6 prevents both primary and secondary nucleation of amyloid and arrests continued assembly of amyloid [22,23,24], it instead could bind along the surfaces or ends of Sup35 amyloid and impair its replication or growth, which would inhibit [*PSI^+^*]^S^ propagation. Due to its limited effect on [*PSI^+^*]^S^, such interactions of DnaJB6b with the type of Sup35 amyloid that propagates as [*PSI^+^*]^S^ might be weaker than those with types of amyloid that underlie [*PSI^+^*]^W^ phenotypes.

Our data correlate the rescue of Sis1JGF cells from [*PSI^+^*]^S^ toxicity to the prevention of hyper-aggregation of NGMC when wild type Sis1 is depleted. Cells transformed by plasmids expressing Sis1, DnaJB6b or DnaJB6b-D33N counteracted toxicity of [*PSI^+^*]^S^ enough to allow growth, while the empty vector or plasmids expressing ST or Sis1-D36N did not. When Sis1 was depleted by addition of doxycycline to [*GPSI^+^*]^S^ cells, the same test proteins that allowed growth of [*PSI^+^*]^S^ strains (Sis1, DnaJB6b and DnaJB6b-D33N) also prevented hyper-aggregation of NGMC upon Sis1 depletion. Likewise, the test proteins that failed to rescue cell growth in [*PSI^+^*]^S^ strains (ST and Sis1-D36N) also failed to prevent hyper-aggregation of NGMC.

Since traces of Sis1 are enough to protect cells from [*PSI^+^*]^S^ toxicity, subtle differences in solubility of Sup35 must be enough to counteract this toxicity. This subtlety suggests propagation of [*PSI^+^*]^S^ is near a threshold of compromising Sup35 function enough to cause harm. Although [*PSI^+^*]^S^ cells lack overt growth phenotypes, they show a stress response [3], which likely reflects the stress of the presence of Sup35 amyloid or this limitation of Sup35 function. This subtlety also likely explains how DnaJB6b can protect cells from [*PSI^+^*]^S^ toxicity without having substantial effects on the prion itself. Supporting this idea are that typical dark pink [*PSI^+^*]^W^ prions, although toxic, are not lethal to Sis1JGF cells, and that strong prions composed of NGMC ([G*PSI^+^*]^S^), whose color phenotype is just slightly pinker than [*PSI^+^*]^S^ prions composed of untagged Sup35 [35], are also toxic but not lethal.

The fact that all cells among replica-plated patches of transformants expressing DnaJB6b or DnaJB6b-D33N grow on FOA after four days, while only rare transformants expressing STC (4–10 colonies/patch of cells) can grow, indicates that the JGF region of DnaJB6b is integral to its efficient function needed to counteract [*PSI^+^*]^S^ toxicity. Since the J domain is not needed for its ability to interact with Hsp70, the benefit of this region is most likely due to the GF portion. This dependency on the GF region represents a deviation from how DnaJB6b protects cells from polyQ toxicity, in which the STC alone works as effectively as wild type DnaJB6b [24]. Continued work to decipher what underlies this difference will provide insight into anti-amyloid activities of DnaJB6 in vivo.

We show that Sis1-D36N has a toxic effect when Sis1 is depleted. In [*psi^−^*] strains, cells can survive without Sis1 if Sis1JGF is present. However, if those cells also contain Sis1-D36N, then they will die. Additionally, cells with their only source of Sis1 regulated by a *TETr* promoter grew like wild type for over 12 generations when Sis1 is depleted with doxycycline, but if the same cells have Sis1-D36N, their growth rate diminished beyond this time. These results indicate that Sis1-D36N can inhibit vital action of both Sis1JGF and the very low amounts of Sis1 remaining after doxycycline treatment, or that Sis1-D36N has a separate toxic effect that requires only a small amount of full length Sis1 to counteract.

DnaJB6 shows promise as a therapeutic [26], yet the considerable difference in its effectiveness on different amyloid structures composed of one and the same protein [24] raises important questions regarding such approaches. Understanding the mechanistic basis underlying these differences will help guide strategies for therapeutic use of DnaJB6. Despite reduced sensitivity of Sup35 amyloid that composes [*PSI^+^*]^S^ to anti-amyloid action of DnaJB6, our system revealed that DnaJB6 can moderate incorporation of Sup35 into this type of amyloid in cells. Continued use of this system should provide additional insights into how DnaJB6b acts to protect cells from the harm associated with cytoplasmic amyloid.

## 5. Conclusions

Our work provides new insights into prions composed of Sup35, toxicity of cytoplasmic amyloid, and cellular factors like DnaJB6b and Sis1 that counteract amyloid toxicity. When function of Sis1 is reduced in [*PSI^+^*]^S^ cells, a subtle reduction in solubility of the essential Sup35 protein due to enhanced depletion by [*PSI^+^*]^S^ prions is enough to make normally benign amyloid-based [*PSI^+^*]^S^ prions lethal. Anti-amyloid activity of DnaJB6b is very effective at curing yeast of [*PSI^+^*]^W^ prions composed of the same Sup35 but is unable to disrupt propagation of [*PSI^+^*]^S^ prions. The toxicity caused by enhanced depletion of Sup35 in [*PSI^+^*]^S^ cells with reduced Sis1 function allowed us to uncover an activity of DnaJB6 that moderates depletion of Sup35 enough to restore viability without noticeably affecting prion phenotype.

Our in vivo [*PSI^+^*]^S^ Sis1JGF system therefore provides a sensitive tool to study protective anti-amyloid effects of DnaJB6b or other protein quality control factors. Based on known activities of DnaJB6b and Sis1, our data prompt sound hypotheses to explain how Sis1 and DnaJB6b can act to protect cells from the presence of amyloid in the cytoplasm, without clearing the amyloid outright. This genetic model system will be useful for future work that can be used to test these hypotheses and to identify interacting factors and molecular interactions that will provide a better understanding of how cells respond to amyloid and protect from its toxic effects.

## Figures and Tables

**Table 1:** Plasmids used in this study.

Plasmid	Marker	Promoter	Protein Expressed	Source
pRS314	*TRP1*	na	empty vector	[36]
pRS315	*LEU2*	na	empty vector	[36]
pYW17	*URA3*	*SIS1*	Sis1	[37]
pMR286	*TRP1*	*GPD*	Sis1-cmyc	[38]
pMR286DN	*TRP1*	*GPD*	Sis1^D36N^-cmyc	[38]
pRED156	*URA3*	*TETr*	Sis1	This study
pMR266	*TRP1*	*GPD*	Sis1	[24]
pMR266DN	*TRP1*	*GPD*	Sis1^D36N^	[24]
JE167	*LEU2*	*GPD*	Sis1-mCh	[19]
JE167DN	*LEU2*	*GPD*	Sis1^D36N^-mCh	This Study
JE237	*LEU2*	*SIS1*	Sis1JGF	This Study
JE65	*TRP1*	*SIS1*	Sis1JGF	This Study
pMR328	*TRP1*	*GPD*	DnaJB6b-cmyc	[24]
pMR328DN	*TRP1*	*GPD*	DnaJB6b^D33N^-cmyc	[24]
pRED155	*TRP1*	*GPD*	DnaJB6b∆ST	[24]
pMR399	*TRP1*	*GPD*	DnaJB6b	This study
pRED152	*TRP1*	*GPD*	DnaJB6b-mCh	This Study
pRED152DN	*TRP1*	*GPD*	DnaJB6b^D33N^-mCh	This Study
pRU14	*LEU2*	*GAL*	DnaJB6b	[24]
pRED114	*TRP1*	*GAL*	DnaJB6b	This study
JE66	*TRP1*	*SIS1*	Sis1	[19]
JE239	*TRP1*	*SIS1*	Sis1	[19]
pRED144	*TRP1*	*GPD*	DnaJB6b-STC	This Study
pRED142	*TRP1*	*GPD*	DnaJB6b-ST	This Study
pRED155	*TRP1*	*GPD*	DnaJB6b∆ST	This Study
pMR329	*TRP1*	*GPD*	DnaJB6b∆STCTD	[24]

**Table 2:** Growth rates of FOA-resistant strains.

Test Protein	Doubling Time (min)	
	[*psi^−^*]	[*PSI^+^*]^S^
Sis1	195 ± 5	225 ± 7
ev (JGF only)	270 ± 10	inviable
JB6	315 ± 5	520 ± 42
JB6-D33N	285 ± 15	455 ± 7

Cells from FOA plates were grown in SC -Leu -Trp medium. All express Sis1JGF from a *LEU2* vector and test protein (left column) from a *TRP1* vector. Values are averages (±range) of two biological replicates.

## Data Availability

Not applicable.

## Supplementary Materials

The following supporting information can be downloaded at: https://www.mdpi.com/article/10.3390/biology11121846/s1, Figure S1: Sis1 does not impair [*PSI^+^*]^S^; DnaJB6b does not change [*PSI^+^*]^S^ prion character. (A) Cells from Figure 3A expressing two copies of Sis1 that show diffuse fluorescence were streaked onto 1/2YPD. All resulting colonies are white with no noticeable red or pink anywhere in the streaks, indicating that all cells giving rise to them propagate [*PSI^+^*]^S^ despite their original [*psi^–^*]-like fluorescence. (B) Cytoduction to infect wild type recipient strain 628-8Cc with prions from red and white cells expressing Sis1 or DnaJB6 (left panel) as indicated. Upper center panel shows plate selecting for 628-8Cc and [*PSI^+^*] prions (cytoductant recipients only), right panel shows selection for diploids regardless of prion status to confirm that cells mated. Patches of cells labeled yellow “C” use control [*PSI^+^*] donor strain—its genotype allows selection for cytoductants but not diploids. 628-8Cc cytoductants from plate in center panel were streaked for colonies onto 1/2YPD to monitor prion phenotype. All have the same prion phenotype. Labels identify the donor source of prions; Figure S2: [*GSPI^+^*]^S^ prions are not lethal. Plasmid shuffle using strain 2140 [*GSPI^+^*]^S^. Cells expressing Sis1JGF only (ev) or Sis1JGF and co-expressing either the ST or STC region of DnaJB6b grow similarly on FOA, indicating ST and STC do not counteract [*GSPI^+^*]^S^ toxicity. Both grow much more slowly than those expressing Sis1 or DnaJB6b (JB6), while those expressing Sis1-D36N (Sis1-DN) do not grow on FOA (note no change in cell density from 2 to 4 days on FOA); Figure S3: A Plasmid shuffle selection on FOA shows fluorescent tags do not alter ability of Sis1 and DnaJB6b test proteins to protect cells from prion toxicity (compare with Figure 1B).
