# Supplementary material for: Human J-Domain Protein DnaJB6 Protects Yeast from [PSI+] Prion Toxicity
Source: Biology (Basel). 2022 Dec 18;11(12):1846. doi: 10.3390/biology11121846 (PMC9776390; doi:10.3390/biology11121846)
Supplement: Supplementary file 1 [file biology-11-01846-s001.zip › Supplementary figure legends.pdf]

Figure S1. Sis1 does not impair [*PSI*$^{+}$]^{S}; DnaJB6b does not change [*PSI*$^{+}$]^{S} prion character. **A** Cells from Figure 3A expressing two copies of Sis1 that show diffuse fluorescence were streaked onto 1/2YPD. All resulting colonies are white with no noticeable red or pink anywhere in the streaks, indicating that all cells giving rise to them propagate [*PSI*$^{+}$]^{S} despite their original [*psi*$^{-}$]-like fluorescence. **B** Cytoduction to infect wild type recipient strain 628-8Cc with prions from red and white cells expressing Sis1 or DnaJB6 (left panel) as indicated. Upper center panel shows plate selecting for 628-8Cc and [*PSI*$^{+}$] prions (cytoductant recipients only), right panel shows selection for diploids regardless of prion status to confirm that cells mated. Patches of cells labeled yellow "C" use control [*PSI*$^{+}$] donor strain – its genotype allows selection for cytoductants but not diploids. 628-8Cc cytoductants from plate in center panel were streaked for colonies onto 1/2YPD to monitor prion phenotype. All have the same prion phenotype. Labels identify the donor source of prions.

Figure S2. [*GSPI*$^{+}$]^{S} prions are not lethal. Plasmid shuffle using strain 2140 [*GSPI*$^{+}$]^{S}. Cells expressing Sis1JGF only (ev) or Sis1JGF and co-expressing either the ST or STC region of DnaJB6b grow similarly on FOA, indicating ST and STC do not counteract [*GSPI*$^{+}$]^{S} toxicity. Both grow much more slowly than those expressing Sis1 or DnaJB6b (JB6), while those expressing Sis1-D36N (Sis1-DN) do not grow on FOA (note no change in cell density from 2 to 4 days on FOA).

Figure S3. **A** Plasmid shuffle selection on FOA shows fluorescent tags do not alter ability of Sis1 and DnaJB6b test proteins to protect cells from prion toxicity (compare with Figure 1B).
