# Supplementary figures and images for: Human J-Domain Protein DnaJB6 Protects Yeast from [PSI+] Prion Toxicity
Source: Biology (Basel). 2022 Dec 18;11(12):1846. doi: 10.3390/biology11121846 (PMC9776390; doi:10.3390/biology11121846)

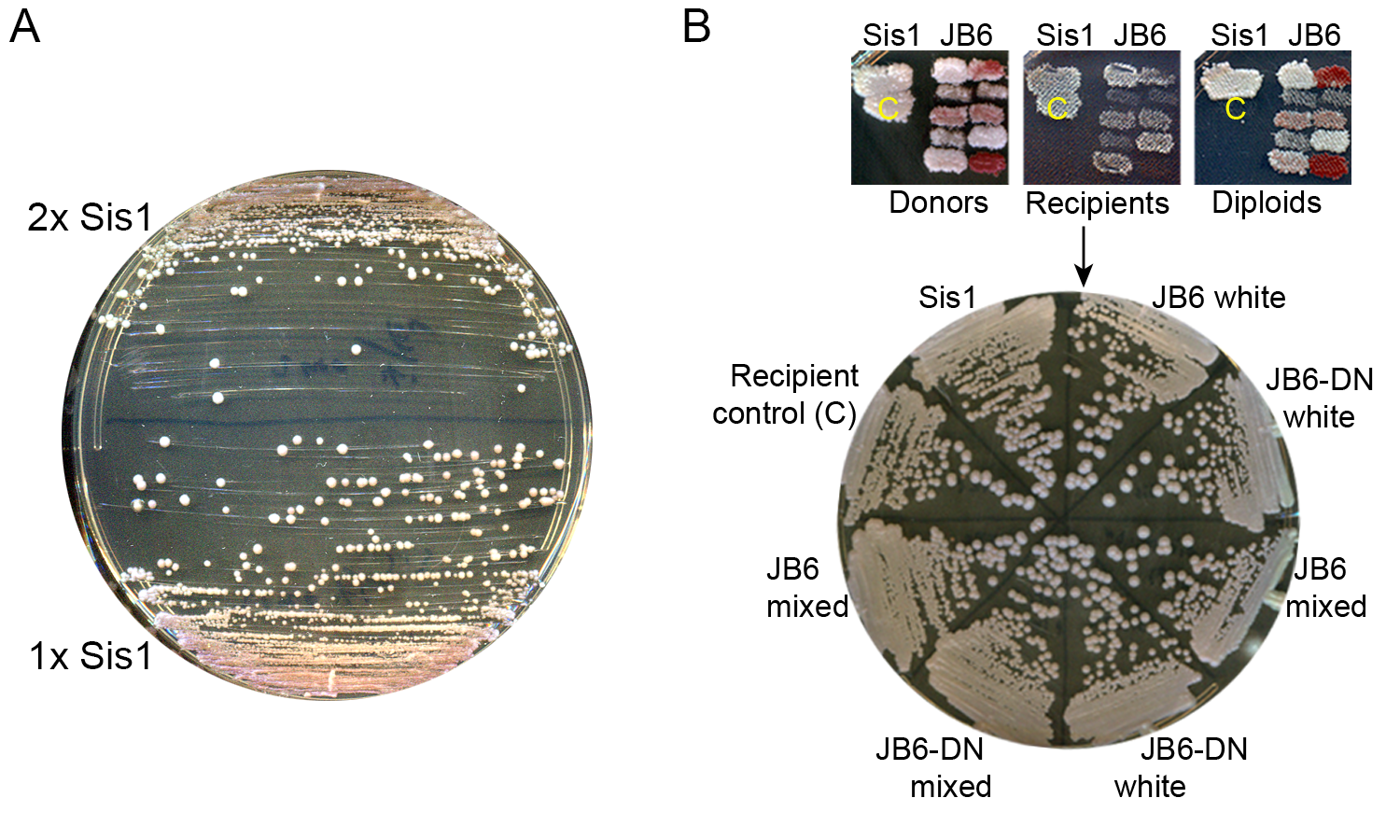

Supplement: Supplementary file 1 [file biology-11-01846-s001.zip › b6psiman Figure S1rev.tif]

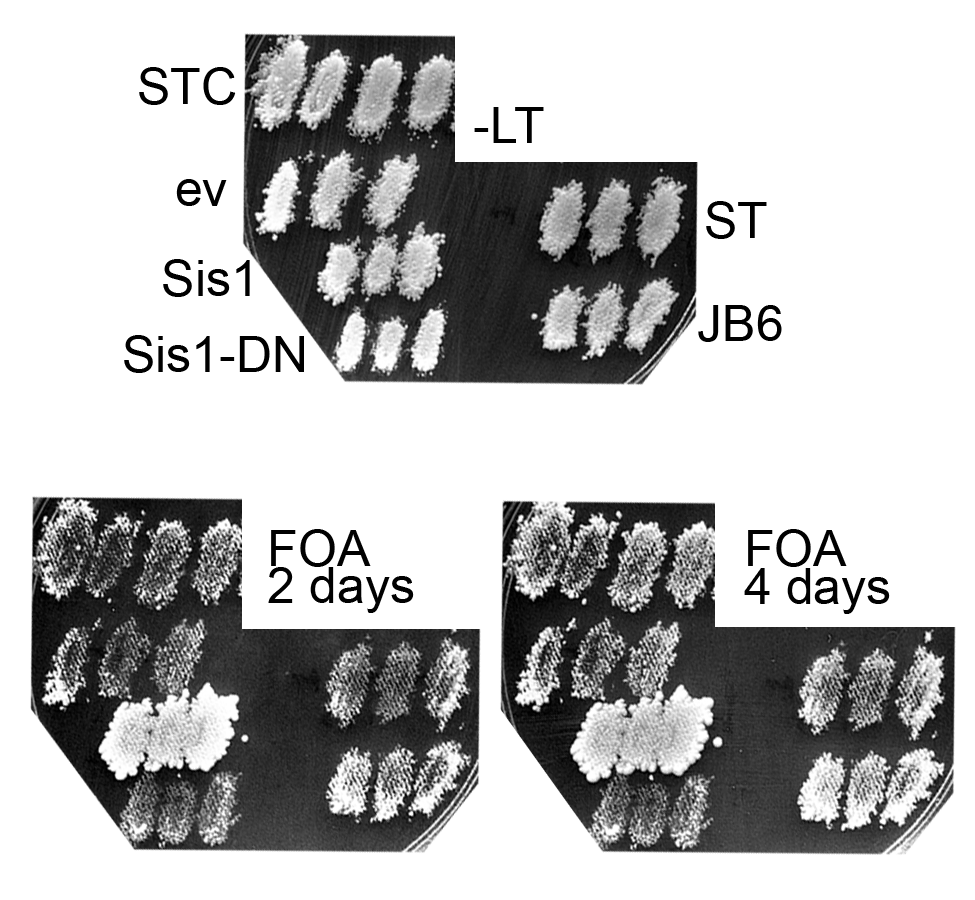

Supplement: Supplementary file 1 [file biology-11-01846-s001.zip › B6psiman Figure S2rev.tif]

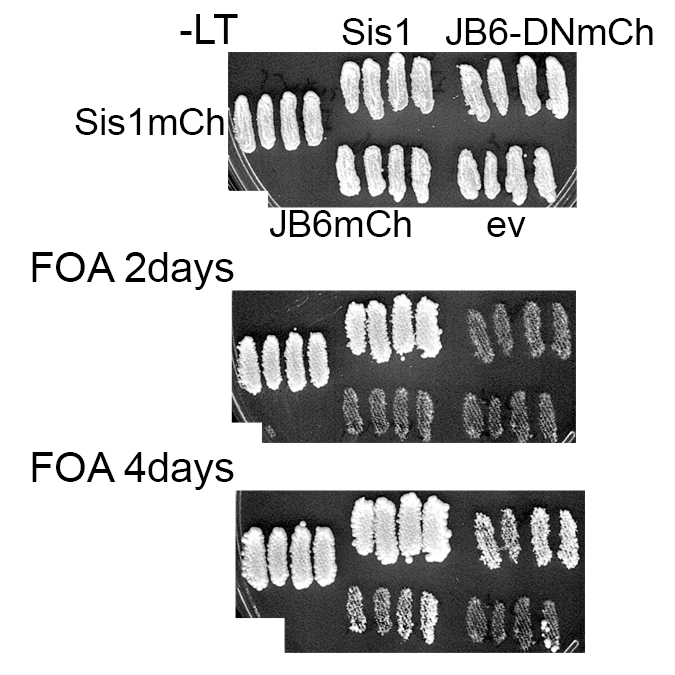

Supplement: Supplementary file 1 [file biology-11-01846-s001.zip › B6psiman Figure S3rev.tif]
